# Association of Blood Lead Levels with Onset of Puberty in Russian Boys

**DOI:** 10.1289/ehp.10516

**Published:** 2008-03-19

**Authors:** Russ Hauser, Oleg Sergeyev, Susan Korrick, Mary M. Lee, Boris Revich, Elena Gitin, Jane S. Burns, Paige L. Williams

**Affiliations:** 1 Environmental and Occupational Medicine and Epidemiology Program, Department of Environmental Health, Harvard School of Public Health, Boston, Massachusetts, USA; 2 Department of Physical Education and Health, Samara State Medical University, Samara, Russia; 3 Chapaevsk Medical Association, Chapaevsk, Samara Region, Russia; 4 Department of Medicine, Brigham and Women’s Hospital, Harvard Medical School, Channing Laboratory, Boston, Massachusetts, USA; 5 Pediatric Endocrine Division, Departments of Pediatrics and Cell Biology, University of Massachusetts Medical School, Worcester, Massachusetts, USA; 6 Center for Demography and Human Ecology of Institute for Forecasting, Russian Academy of Sciences, Moscow, Russia; 7 Department of Biostatistics, Harvard School of Public Health, Boston, Massachusetts, USA

**Keywords:** children, environment, epidemiology, lead, puberty

## Abstract

**Background:**

Epidemiologic studies suggest a temporal trend of earlier onset and longer duration of puberty, raising concerns regarding the potential impact of environmental factors on pubertal development. Lead exposure has been associated with delayed pubertal onset in girls; however, epidemiologic data in boys are limited.

**Methods:**

We used multivariable logistic regression models to explore the cross-sectional association of blood lead levels with growth and pubertal onset based on physician-assessed testicular volume (TV) and pubertal staging in 489 boys 8–9 years of age from Chapaevsk, Russia. We used multivariable linear regression models to assess associations of blood lead levels with somatic growth at the study entry visit.

**Results:**

The median (25th–75th percentile) blood lead level was 3 μg/dL (2–5 μg/dL). Height, weight, body mass index, birth weight, and gestational age were predictive of the onset of puberty as assessed either by TV (> 3 mL), genitalia stage (G2), or both. Blood lead level was inversely associated with height (*p* < 0.001) and weight (*p* = 0.06) after adjustment for birth weight, gestational age, and age at examination. In multivariable adjusted analyses, boys with blood lead levels ≥ 5 μg/dL had 43% reduced odds of having entered G2 compared with those with lower levels (odds ratio = 0.57; 95% confidence interval, 0.34–0.95, *p* = 0.03).

**Conclusions:**

Relatively low environmental blood lead levels were associated with decreased growth and differences in pubertal onset in periadolescent Russian boys. Future analyses of this prospective cohort will address pubertal onset and progression in relation to lead and other environmental chemicals.

Puberty, the period of transition from childhood to attainment of mature reproductive function, is characterized by accelerated growth, development of secondary sexual characteristics, and psychological changes. Activation of the hypothalamic–pituitary–gonadal axis, manifest as increasing pulsatile secretion of gonadotropin-releasing hormone (GnRH), initiates puberty. Although the precise mechanisms responsible for pubertal onset are not fully understood, body mass and nutritional status (Vizmanos and Marti-Henneberg 2000), psychosocial health, genetic factors, and neuroendocrine inputs to the hypothalamus are all important determinants (Nathan and Palmert 2005).

Epidemiologic studies suggest a temporal trend of earlier pubertal onset in girls (Anderson et al. 2003; Herman-Giddens et al. 1997), with less consistent evidence in boys (Herman-Giddens et al. 2001; Karpati et al. 2002). Despite the earlier onset, the age of attainment of sexual maturity has not changed. These temporal shifts have raised concerns regarding the potential impact of environmental factors on pubertal development. In girls, the Third National Health and Nutrition Examination Survey (NHANES III), 1988–1994, showed cross-sectional associations of blood lead levels with delayed breast and pubic hair development and menarche (Selevan et al. 2003; Wu et al. 2003). In boys, several studies report a relationship between lead exposure and diminished early childhood growth (Ballew et al. 1999; Kafourou et al. 1997; Schwartz et al. 1986; Vivoli et al. 1993), but we are not aware of published data on its impact on puberty.

The present study was conducted among boys from Chapaevsk, a town of 72,000 residents, in central Russia. Several large industries in Chapaevsk previously manufactured chemical warfare agents but presently produce industrial and agricultural chemicals (Revich et al. 2001). These processes have caused environmental contamination with dioxins and metals, including lead. Additional potential sources of lead exposure are leaded gasoline (Rubin et al. 2002) and lead solder in plumbing. We designed a longitudinal cohort study to explore the impact of environmental contaminants on growth and sexual maturation among Russian boys in Chapaevsk. Boys 8–9 years of age were recruited as subjects of the study to examine the impact of environmental toxicants on both pubertal onset and progression. In this article we present the results of the cross-sectional analysis of the baseline data at 8–9 years of age.

## Methods

### Informed consent

The study was approved by the Human Studies Institutional Review Boards of the Chapaevsk Medical Association, Harvard School of Public Health, University of Massachusetts Medical School, and Brigham and Women’s Hospital. The parent or guardian signed an informed consent, and the boy signed an assent form.

### Study population

All boys 8–9 years of age who were residents of Chapaevsk, Russia, between May 2003 and May 2005 were identified, using health insurance records and the centralized clinic system servicing all Chapaevsk children. The following were exclusion criteria: *a*) children without legal guardians (e.g., children in orphanages), because birth or family history was unavailable; *b*) children of Azerbaijanian nationality, because they were generally born elsewhere and likely to relocate; and *c*) children with chronic illnesses that might affect growth and puberty, such as severe asthma, renal failure, diabetes, and congenital cardiac defects.

The primary caregiver (mother for 448 boys, father for 6, and grandmother, other relative, or foster parent for 35) completed nurse-administered health, lifestyle, and dietary questionnaires. The health and lifestyle questionnaires were developed through pilot work in Chapaevsk (Hauser et al. 2005; Lee et al. 2003) and were revised accordingly to be culturally relevant. The semiquantitative food frequency questionnaire (FFQ) was modified from the validated Russian Institute of Nutrition (RIN) FFQ (Martinchik et al. 1998). Macronutrients were computed from dietary intake using the RIN nutrient database. Birth weight and gestational age were obtained from medical records unless unavailable (3% and 36% of boys, respectively), in which case questionnaire data were used.

With a nurse present, a trained physician (O.S.) performed a standardized examination including height, weight, and pubertal staging without knowledge of the boys’ lead levels. Height (to the nearest 0.1 cm) was measured in stocking feet using a stadiometer. Weight (to the nearest 0.1 kg) was measured with the boys in undergarments using a balance scale with moveable weights and level platform.

Pubertal status was staged from 1 to 5 by visual inspection and compared with published photographs according to internationally accepted criteria (Marshall and Tanner 1970). Pubarche (P, pubic hair stage) was determined by extent of terminal pubic hair growth. Genital staging (G) was assessed by genital size and maturity. Testicular volume (TV) was measured using a Prader orchidometer (Genentech, Inc., San Francisco, CA, USA), and stretched phallic length was measured to the nearest 1.0 mm using a firm ruler. Pubertal onset was defined in two ways: stage G2 or greater, or TV > 3 mL of either testis.

### Blood lead analysis

At the physical examination visit, 3.0 mL of venous blood was collected in trace metal–free Vacutainer tubes (Becton-Dickinson, Franklin Lakes, NJ, USA) after cleaning the site serially with three alcohol pads. Whole blood samples were diluted with a matrix modifier solution and analyzed by Zeeman background-corrected flameless graphite furnace atomic absorption (ESA Laboratories, Inc., Chelmsford, MA, USA). All measured values were reported. The detection limit was 1.0 μg/dL.

### Statistical analysis

Although the data are from a prospective cohort study on the growth and development of Russian children, for this study only the baseline measurements for exposure were available. Therefore, the statistical analyses explored the cross-sectional association of blood lead levels with measures of growth and pubertal onset at baseline. Blood lead levels were modeled as either a continuous measure based on a natural logarithmic transformation to approximate a normal distribution or a dichotomous indicator of high lead defined as ≥ 5 μg/dL. We evaluated the association of log-transformed lead levels with somatic growth [height, weight, and body mass index (BMI, weight in kilograms/squared height in meters)], penile length, and birth characteristics (birth weight and gestational age) using both univariate and multivariable linear regression models adjusted for potential confounders (birth weight, gestational age, and age at examination). All statistical models used the exact age of each boy in days based on the actual birth date. Study visits were timed to be close to the birthday, and the majority were conducted within 1 month of each boy’s birth date. Standard diagnostic methods were used to evaluate the appropriateness of the linear regression model (e.g., homoscedasticity) and to identify potential outliers.

To evaluate the association of both log-transformed lead and high lead (≥ 5 μg/dL) with pubertal onset, we used logistic regression models for the binary outcomes of TV > 3 mL and ≥ stage 2 for genitalia and pubarche, considered separately. We also fit ordinal logistic regression models under a proportional odds assumption for TV (range, 1–6). A test of the proportional odds assumption was conducted for each model, and there was no evidence that this assumption was violated. Similar to the above linear regression models, the logistic regression models were first fit to evaluate univariate effects of log-transformed lead or high lead, and then adjusted for birth weight, gestational age, height, BMI, and age at examination. Selection of covariates in the multivariable models were based on the association of birth history and somatic characteristics with puberty onset in the absence of lead. In addition, an assessment of potential confounding by nutritional status of the associations between lead and pubertal onset was conducted based on preliminary dietary information. Multivariate regression models were further adjusted for macronutrients (protein, carbohydrate, total fat) as a percentage of caloric intake, using the nutrient density method (Willett et al. 1997). A sensitivity analysis of the influence of high blood levels on associations with pubertal onset was conducted by excluding 16 (3%) subjects with blood lead values of ≥ 10 μg/dL.

## Results

Demographic data and birth characteristics of the 489 boys enrolled in the study with blood lead levels, physical examinations, and questionnaire information are presented in Table 1. The boys had a mean (± SD) BMI of 15.9 ± 2.3, similar to the mean BMI of 15.8 at 8 years and 16.2 at 9 years in U.S. children [Centers for Disease Control and Prevention (CDC) 2000]. Preliminary dietary analysis indicated that the estimated proportion of calories from protein (11.6%), carbohydrates (54.0%), and total fats (34.4%) in these Russian boys was similar to those in U.S. boys 6–11 years of age (12.7, 55.2, and 32.6%, respectively) (using NHANES 1999–2000 for comparison; Wright el al. 2003).

**Figure 1:**
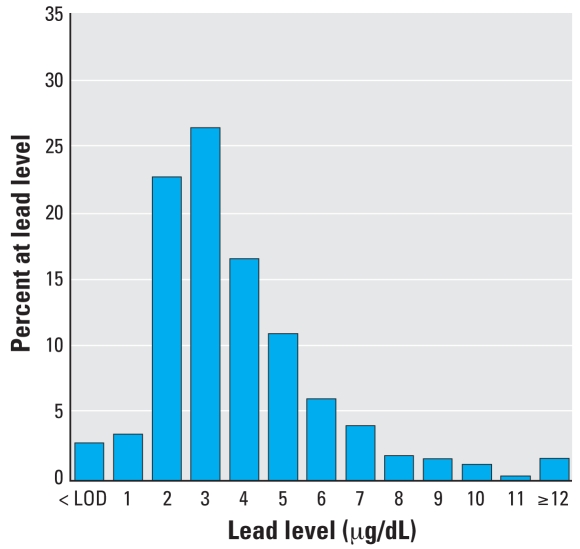
Distribution of blood lead levels (μg/dL) among 489 Russian boys 8–9 years of age. LOD, limit of detection.

The median (25th–75th percentile) blood lead levels of the boys were 3 μg/dL (2–5 μg/dL), with slightly higher levels among 9-year-old boys compared with 8-year-olds (Table 1). Twenty-eight percent of the boys had blood lead levels ≥ 5 μg/dL, with 3% > 10 μg/dL. Two boys had blood lead levels of 30 and 31 μg/dL, and 3% had levels below the limit of detection (1.0 μg/dL). The distribution for blood lead was skewed, necessitating log transformation for statistical analysis (Figure 1).

Height and weight, but not BMI or penile length, were inversely associated with blood lead concentrations (Table 2). For each log unit increase in blood lead level, height decreased 1.42 cm and weight decreased 0.76 kg, on average (*p* < 0.001 and *p* = 0.06, respectively).

Pubertal onset defined as TV > 3 mL was present in 14% of the boys, including 12% of 8-year-olds and 18% of 9-year-olds (Table 1). Pubertal onset, defined by G2 or greater, was present in 30% of all boys but was more common in 9-year-olds [43%; 95% confidence interval (CI), 35–50%] than in 8-year-olds (22%; 95% CI, 18–27%). Stage 2 or greater for pubarche (P2) was present in 7% of boys, 3% of 8-year-olds, and 13% of 9-year-olds. None of the boys were in stage G3 or P3 or greater.

Height, weight, BMI, birth weight, and gestational age were predictive of pubertal onset as defined by either TV > 3 mL or G2, or both. In multivariable models with adjustment for exact age at examination and other growth and birth history measures, there were almost twice the odds of being in G2 per kilogram increase in birth weight [odds ratio (OR) = 1.91; 95% CI, 1.15–3.17; *p* = 0.01] and a 13% increase in the odds of being in G2 per one-unit increase in BMI (OR = 1.13; 95% CI, 1.03–1.23; *p* = 0.01). Similar magnitudes of associations were found for these predictors and pubertal onset using TV categorized as six levels (1–6 mL) and two levels (≤ 3 mL and > 3 mL). Gestational age was inversely associated with pubertal onset based on TV > 3 mL (OR = 0.76; 95% CI, 0.63–0.93; *p* = 0.006).

After adjustment for confounders, when pubertal onset was defined using G2, each log unit increase in blood lead concentration was associated with a marginally significant reduction in odds of being in G2 (OR = 0.75; 95% CI, 0.53–1.06; *p* = 0.10) (Table 3). When blood lead level was dichotomized at 5 μg/dL, those with high blood lead (≥ 5 μg/dL) had a statistically significant 43% reduced odds of being in G2 (OR = 0.57; 95% CI, 0.34–0.95; *p* = 0.03). In unadjusted models, there was an association between TV (which ranged from 1 to 6 mL) with dichotomized blood lead (OR = 0.57; 95% CI, 0.39–0.83; *p* = 0.004). However, after adjustment for covariates, the association became only marginally significant (OR = 0.72; 95% CI, 0.48–1.07; *p* = 0.11). No association of blood lead levels with either TV > 3 mL or stage P2 was identified, despite trends in the same direction as G2.

Several sensitivity analyses were conducted to evaluate the impact of nutritional status, socioeconomic status, a higher blood lead cut point (≥ 10 μg/dL), and elimination of outliers on the robustness of the associations between blood lead and pubertal onset. These sensitivity analyses helped to confirm and support the previous results. Associations of blood lead with G2 had similar ORs but narrower CIs after further adjustment for dietary macronutrients using the nutrient density approach in models with both log lead (OR = 0.70; 95% CI, 0.49–1.01; *p* = 0.06) and high blood lead (OR = 0.52; 95% CI, 0.31–0.88; *p* = 0.01). In addition, the association of TV with high lead (≥ 5 μg/dL) became significant after adjustment for dietary macronutrients (OR = 0.66; 95% CI, 0.44–1.00, *p* = 0.05).

In the multivariate models described above, both parental education and household income levels were evaluated as potential confounders of the relationship between puberty onset and blood lead. Household income level was associated with both blood lead levels and with puberty onset in univariate analysis, suggesting that it could be a potential confounder. However, after adjustment by household income in the models presented in Table 3, there was no evidence of confounding by household income in models using either log-transformed lead or high lead. The associations presented in Table 3 were essentially unchanged by including household income. Parental education level was inversely associated with lead levels but was not associated with pubertal onset; thus it was not expected to be a confounder. Including parental education level in all adjusted models shown in Table 3 did not change effect estimates of the associations of blood lead with pubertal onset. Similarly, adjustment for weight had essentially no effect on estimated associations of blood lead with pubertal onset after adjustment for other similar growth measures (height, BMI).

Because shorter boys may have higher lead levels than taller boys and are less likely to have puberty onset, we also conducted several analyses to specifically assess the impact of stature on associations between blood lead levels and puberty. The adjusted models in Table 3 suggest an association between G2 and high lead levels, even after controlling for actual height. In addition, a stratified logistic regression model, stratifying by height (< 127 cm, 127–133 cm, > 133 cm), yielded adjusted results similar to those in Table 3 for G2 with log lead (OR = 0.74; 95% CI, 0.53–1.05, *p* = 0.09) and with high lead (OR = 0.58; 95% CI, 0.35–0.96; *p* = 0.03).

After excluding 16 subjects with blood lead values ≥ 10 μg/dL, the significant association of pubertal onset reflected by G2 with high versus low blood lead persisted (OR = 0.60; 95% CI, 0.35–1.02, *p* = 0.06). Similarly, exclusion of two boys with lead levels ≥ 30 μg/dL had essentially no effect on the results shown in Table 3. In addition, we evaluated the association of G2 with an alternate cutoff for high lead defined as ≥ 10 μg/dL. Although the percentage of boys with high blood lead defined as such was small (3%), limiting the statistical power for identifying associations, the magnitude and direction of the association with G2 was similar to that shown in Table 3 (OR = 0.49 compared with OR = 0.57 for the 5-μg/dL cutoff).

## Discussion

In this cohort of Russian boys, blood lead levels were inversely associated with height, weight, and pubertal onset defined as pubertal stage G2 or greater. The association with pubertal onset was strongest when blood lead was dichotomized at 5 μg/dL. Boys with blood lead ≥ 5 μg/dL had a 44% reduced odds of having entered G2. These findings are consistent with previously described cross-sectional associations of blood lead with delayed pubertal onset in U.S. girls with lead levels below the CDC definition of childhood lead poisoning (< 10 μg/dL or 0.48 μmol/L) (CDC 1991; Selevan et al. 2003; Wu et al. 2003). In addition, dose–response extrapolations using rodent models of lead-associated delays in male sexual development (Ronis et al. 1998b) estimate a no observed effect level threshold of 3 μg/dL for delayed pubertal onset, an exposure threshold well within background population lead exposures and consistent with our findings. Despite the downward trend in blood lead levels, a large number of U.S. children still have values > 10 μg/dL, and an even larger number have levels between 5 and 10 μg/dL (CDC 2005).

Our findings suggest that lead is unlikely to be playing a role in the observed secular trend of earlier pubertal onset. However, independent of lead exposure, the pattern of pubertal development among the Chapaevsk boys (Lee et al. 2003) parallels NHANES III data showing earlier onset but no change in the age of attainment of sexual maturity, suggesting that although the onset of puberty may be earlier, the tempo is slower (Herman-Giddens et al. 2001; Sun et al. 2002). For example, among non-Hispanic white boys in NHANES III, 1988–1994, 29% of 8-year-olds and 36% of 9-year-olds had entered G2 (Herman-Giddens et al. 2001). Similarly, among this cohort of Russian boys, 22% of 8-year-olds and 43% of 9-year-olds had entered G2. These observations are consistent with our previous cross-sectional study of 2,579 boys between 10 and 16 years of age in Chapaevsk, which reported that 79% of 10-year-old boys were already in G2 or greater (Lee et al. 2003). Despite this earlier onset of puberty, only 18% of 15-year-olds and 22% of 16-year-olds had achieved stage G5, indicating that attainment of sexual maturity was not accelerated.

Among these Russian boys, there was a higher prevalence of pubertal onset as defined by ≥ G2 (30%) compared with that defined by TV > 3 mL (14%). Of the boys in G2, 60% had a prepubertal TV, whereas of the boys with TV > 3 mL, all but 12 boys (17%) were in G2. Although changes in genital staging and TV are thought to occur in parallel, few studies have specifically assessed both of these signs of pubertal onset. A similar discordance has been reported in a large East German study in which the 3rd percentile for G2 occurred at 8.5 years in contrast to 11.8 years for achieving a testicular length ≥ 30 mm (in early puberty, comparable to 3 mL) (Willers et al. 1996). Our inclusion of both measures enabled us to detect small differences in their association with blood lead levels. The stronger relation of blood lead with G2 compared with TV may reflect either differential sensitivities of genital staging and TV to low-level lead exposure or increased power to detect effects resulting from the higher prevalence of G2, or both. As this study population ages and proportionately more boys enter puberty, we will have increased power to determine whether lead has a differential effect on puberty measured by genital staging versus TV or on the tempo of puberty.

Animal models suggest that both the timing and duration of exposure may be important for lead’s reproductive toxicity. For example, male rats exposed to lead during gestation had decreased birth weights and pubertal growth rates due to disrupted growth hormone (GH) secretion and decreased plasma insulin-like growth factor 1 (IGF-1) concentrations during puberty (Ronis et al. 1996, 1998a). Similarly, in lead-exposed children, reduced 24-hr GH levels, IGF-1, and stimulated GH release have also been reported (Huseman et al. 1987, 1992).

Chronic lead exposure from gestation through puberty resulted in delayed sexual maturation of male rats measured by prostate weight and secondary sex organ development (Ronis et al. 1998b). In that model, lead exposure was associated with lower plasma testosterone and luteinizing hormone (LH) concentrations and elevated pituitary LH content at puberty. Lead exposure has also been shown to suppress serum testosterone levels and spermatogenesis without a significant change in circulating gonadotropins (Ronis et al. 1996; Sokol 1987; Sokol et al. 1985). Long-term low-dose exposure to lead (at blood levels as low as 10 μg/dL) induced the expression of hypothalamic GnRH mRNA in male rats, whereas peripheral concentrations of GnRH and LH were not significantly altered, suggesting a disruption of hypothalamus–pituitary signaling (Sokol et al. 2002). The authors hypothesized that lead interfered with GnRH release, an observation supported by autoradiographic studies showing the greatest ^210^Pb accumulation in the median eminence (Stumpf et al. 1980).

Interestingly, birth weight and gestational age were associated with onset of puberty. For each kilogram increase in birth weight, there were almost twice the odds of being in G2 and similar magnitudes of associations for pubertal onset using TV categorized as six levels. Gestational age was inversely associated with pubertal onset based on TV > 3 mL. These results suggest that there is an early developmental component to puberty. However, there is limited evidence for an association of perinatal factors and puberty onset in boys (Persson et al. 1999). Although not confirmed by others, one study has show an association of intrauterine growth retardation in girls with an earlier pubertal onset and menarche (Ibanez and de Zegher 2006).

Several uncertainties and limitations are associated with the cross-sectional design of the present study. For example, we cannot assess the relationship of prenatal or chronic lead exposure with pubertal onset; thus, further exploration of patterns of lead exposure in this population are warranted. A further limitation is that the 8- to 9-year-old boys in the present analysis are younger than the median age of pubertal milestones for genitalia, pubic hair, and testicular development (Karlberg 2002). This may result in the present study being underpowered to detect associations of these pubertal milestones with lead. The pubertal data on boys 8–9 years of age in the present article are baseline data from an ongoing prospective cohort study on pubertal development. Follow-up data will be available in the coming years to more fully explore these preliminary associations as well to investigate the association of lead with progression of puberty, controlling for changes in growth over time. Because of the paucity of epidemiologic data on the association of lead with pubertal onset in boys, the present analyses make a contribution to the literature and suggest further areas of study.

Faster physical growth and development during adolescence leads to increased volume of distribution, particularly bone, and may result in lower blood lead levels (O’Flaherty 1995). This potential reverse causality, however, is unlikely in the study age group (8–9 years of age), given that accelerated pubertal growth in boys is typically observed at stages 3 and 4 of pubertal maturation rather than at the onset. In addition, the association of high lead with G2 persisted even after adjustment for stature and after stratification by height levels.

In addition to lead, dioxin exposure is of concern for the Russian boys studied. In preliminary analyses on data from 125 boys, serum dioxin had no correlation with blood lead levels (*r* = 0.06; *p* = 0.5), suggesting that dioxin exposure is unlikely to confound our findings. In future work, we will complete dioxin analyses for the remaining study participants and explore the interrelationships of lead and dioxin with growth and development.

In conclusion, to the best of our knowledge, this is the first epidemiologic study to demonstrate a relationship between blood lead levels and reduced growth and sexual maturation in periadolescent boys. Most importantly, the association of blood lead levels with growth and pubertal onset occurred at a relatively low level (≥ 5 μg/dL). Although the lead-associated changes in pubertal onset of the boys in the study do not constitute clinically defined pubertal delay, modest changes in the mean age of pubertal onset can result in substantial increases in the occurrence of clinically abnormal onset in a population, and thereby have important public health consequences (Bellinger et al. 2007). Because a large number of children in the general population have blood lead levels within the range of the study, these observations raise concerns regarding the potential consequences for population-wide alterations in male pubertal timing.

## Figures and Tables

**Table 1:** Demographics, growth and sexual maturation characteristics, and blood lead levels among Russian boys 8–9 years of age.

Characteristic	Boys 8 years of age (*n* = 304)	Boys 9 years of age (*n* = 185)	Total boys (*n* = 489)
Growth and sexual maturation parameters			
Age [years (mean ± SD)]	8.04 ± 0.10	9.02 ± 0.08	8.41 ± 0.49
Height [cm (mean ± SD)]	128.42 ± 5.91	133.00 ± 5.96	130.16 ± 6.32
Weight [kg (mean ± SD)]	26.49 ± 5.47	28.37 ± 5.65	27.20 ± 5.61
BMI (mean ± SD)	15.94 ± 2.30	15.94 ± 2.34	15.94 ± 2.31
Penile length [cm (mean ±SD)]^a^	5.49 ± 0.78	5.37 ± 0.67	5.45 ± 0.74
TV (mL) [no. (%)]^b^			
1	8 (3)	4 (2)	12 (2)
2	151 (50)	80 (44)	231 (48)
3	108 (36)	66 (36)	174 (36)
4	28 (9)	22 (12)	50 (10)
5	5 (2)	8 (4)	13 (3)
6	3 (1)	2 (1)	5 (1)
TV > 3 mL [no. (%)]^b^	36 (12)	32 (18)	68 (14)
Tanner staging [no. (%)]			
Genitalia			
G1	237 (78)	107 (58)	344 (70)
G2	67 (22)	78 (42)	145 (30)
Pubarche			
P1	293 (96)	154 (83)	447 (91)
P2	11 (4)	31 (17)	42 (9)
Birth history			
Birth weight [kg (mean ± SD)]^c^	3.34 ± 0.54	3.33 ± 0.52	3.34 ± 0.53
Gestational age [weeks (mean ± SD)]^d^	38.96 ± 1.88	39.15 ± 1.54	39.03 ± 1.76
Household characteristics			
Family income (rubles/month) [no. (%)]^e^			
< 2,200	16 (5)	15 (8)	31 (6)
2,200–3,599	33 (11)	33 (18)	66 (14)
3,500–4,999	41 (13)	35 (19)	76 (16)
5,000–7,200	71 (24)	56 (30)	127 (26)
> 7,200	142 (47)	46 (25)	188 (38)
Mother’s age at son’s birth (mean ± SD)^f^	24.2 ± 5.3	23.6 ± 4.8	24.0 ± 5.1
Blood lead levels (μg/dL)			
Median (25th–75th percentile)	3 (2–4)	4 (3–5)	3 (2–5)
0–2 [no. (%)]	103 (34)	39 (21)	142 (29)
3–4 [no. (%)]	129 (43)	81 (44)	210 (43)
≥ 5 [no. (%)]	72 (24)	65 (35)	137 (28)

a Four missing penile length.

b Maximum of left and right TV; four missing TV.

c Three missing birth weight.

d Four boys missing gestational age.

e One missing household income,

f Five missing age of mother at birth of son.

**Table 2:** Association of lead (natural log transformation) with measures of physical growth and birth characteristics among Russian boys 8–9 years of age (*n* = 489), based on univariate and multiple linear regression. models.

	Unadjusted regression coefficient			Adjusted regression coefficient^a^		
Characteristic	Estimate	95% CI	*p*-Value	Estimate	95% CI	*p*-Value
Height (cm)	−1.043	−1.95 to −0.13	0.02	−1.439	−2.25 to −0.63	< 0.001
Weight (kg)	−0.764	−1.57 to 0.04	0.06	−0.761	−1.54 to 0.02	0.067
BMI	−0.206	−0.54 to 0.13	0.22	−0.107	−0.44 to 0.23	0.53
Penile length (cm)	−0.004	−0.11 to 0.10	0.94	0.023	−0.09 to 0.13	0.68
Birth weight (kg)	−0.094	−0.17 to −0.02	0.02	−0.084	−0.15 to −0.02	0.01
Gestational age (weeks)	−0.038	−0.29 to 0.22	0.77	0.118	−0.09 to 0.33	0.27

a Linear regression models for the outcomes height, weight, BMI, and penile length were adjusted for birth weight, gestational age, and age at exam. The model for birth weight was adjusted for height, weight, BMI, penile length, and gestational age. The model for gestational age was adjusted for height, weight, BMI, penile length, and birth weight.

**Table 3:** Odds ratios for the association of lead (natural log transformation and high lead) with puberty onset among Russian boys 8–9 years of age (*n* = 489), based on logistic regression models.

	Unadjusted regression coefficient		Adjusted regression coefficient^a^	
Model	OR (95% CI)	*p*-Value	OR (95% CI)	*p*-Value
Models for effect of lead (natural log transformation) with puberty onset				
TV				
All six levels^b^	0.80 (0.61–1.06)	0.12	0.90 (0.67–1.20)	0.47
Puberty onset (volume > 3 mL)	1.01 (0.67–1.53)	0.96	1.08 (0.69–1.70)	0.74
Tanner staging				
Tanner stage ≥ G2	0.82 (0.60–1.12)	0.20	0.75 (0.53–1.06)	0.10
Tanner stage ≥ P2	1.37 (0.81–2.33)	0.24	1.08 (0.60–1.93)	0.81
Models for effect of high lead (≥ 5 μg/dL) with puberty onset				
TV				
All six levels^b^	0.57 (0.39–0.83)	0.004	0.72 (0.48–1.07)	0.11
Puberty onset (volume > 3 mL)	0.77 (0.42–1.40)	0.39	0.83 (0.43–1.59)	0.58
Tanner staging				
Tanner stage ≥ G2	0.58 (0.36–0.92)	0.02	0.57 (0.34–0.95)	0.03
Tanner stage ≥ P2	0.90 (0.44–1.85)	0.78	0.74 (0.34–1.60)	0.44

a Adjusted for birth weight, gestational age, height, BMI, and age at examination.

b Based on ordinal logistic regression model under proportional odds assumption.
